# Identification of genes promoting fitness of a plant-associated *Salmonella* Choleraesuis strain on alfalfa sprouts during cold storage

**DOI:** 10.1128/aem.00604-26

**Published:** 2026-08-10

**Authors:** Maximilian Beck, Laura Führer, Steffen Porwollik, Weiping Chu, Verena Hohenester, Irmak Sah, Michael McClelland, Claudia Guldimann, Irene Esteban-Cuesta

**Affiliations:** 1 Competence Center for Food Safety, Chair of Food Safety and Analytics, Veterinary Faculty, LMU Munich, Oberschleissheim, Germany; 2 Department of Microbiology and Molecular Genetics, School of Medicine, University of California, Irvine, California, USA

**Keywords:** transposon insertion sequencing, salmonellosis, microbial contamination, transposon library, food safety

## Abstract

Consumption of sprouted seeds, such as alfalfa sprouts, has increased in recent years due to their nutritional value and antioxidant content. However, these products have repeatedly been implicated in outbreaks of foodborne pathogens, including *Salmonella enterica*. Although host-adapted *Salmonella* serovars are less frequently associated with foodborne illness, infections caused by these serovars often result in invasive and severe outcomes, highlighting the importance of understanding their persistence in food production systems. Moreover, the variability among *Salmonella* serovars requires characterization beyond the most prevalent types to support the development of precision food safety strategies effective across the diversity of serovars capable of contaminating fresh produce. Here, a plant-internalized *Salmonella* Choleraesuis strain was used as a model to investigate persistence mechanisms on alfalfa sprouts. A bar-coded transposon mutant library comprising approximately 33,000 unique insertions was generated, along with a collection of individual insertion mutants. These resources were used to identify genetic determinants contributing to strain fitness on sprouts under abusive cold storage (8°C) simulating commercial shelf-life environments. Genome-wide analyses identified negative selection for mutants with insertions in *eda*, *fabF*, *lpp1_2*, *pnp*, *stpA*, *SCHChr_03621*, and two intergenic regions. Competition assays confirmed fitness defects associated with *eda*, encoding a key enzyme of the Entner-Doudoroff pathway; *mnmG*, encoding a tRNA modification enzyme involved in translational fidelity; and *fabF*, involved in fatty acid biogenesis. These findings provide a genome-wide perspective on mechanisms enabling persistence on sprouts of a plant-associated, host-adapted *Salmonella* strain during cold storage and inform risk assessment and intervention design within precision food safety frameworks.

Sprouts are germinated seeds that are harvested before true leaves develop and generally consumed raw (1). Due to their high nutrient concentration and antioxidant content, sprouts are perceived as healthy foods, and their consumption has increased (2). However, the warm, humid production environment, together with the release of readily available nutrients such as sugars and amino acids during seed germination, creates favorable conditions for microbial proliferation (3). As a result, sprouts are frequently associated with elevated microbial loads, including a notable prevalence of antimicrobial-resistant bacteria (4, 5).

Multiple foodborne outbreaks have been linked to contaminated sprouted seeds, specifically involving *Escherichia coli* O157:H7 (6) and several *Salmonella enterica* serovars (7). *S. enterica* is of particular concern due to its significant global public health impact (8) and frequent involvement in sprout-associated outbreaks (3, 9, 10). This highlights the ability of *S. enterica* to persist throughout the entire sprout production and distribution system, ultimately reaching consumers.

Previous studies have explored epidemiologically dominant serovars, including attachment mechanisms of *S.* Newport and Typhimurium to sprouts during the germination process at room temperature (11, 12), as well as the interactions of Typhimurium and Enteritidis, under conditions that mimic the shelf life of sprouted seeds under refrigeration. Although common persistence-associated pathways have been identified across multiple *Salmonella* serovars, their relative contribution to fitness has been shown to vary with the serovar, food matrix, and environmental conditions, highlighting the need to investigate a broader range of serovars in food-associated settings. The broad diversity among *S. enterica* serovars contributes to differences in environmental persistence, host range, and disease severity (13), emphasizing the need to characterize survival mechanisms beyond the most frequently reported serovars.

In this study, we investigate the genetic determinants required for the fitness of an *S. enterica* serovar Choleraesuis isolate during abusive cold storage on alfalfa sprouts. A storage temperature of 8°C was selected to reflect a realistic temperature abuse scenario, as temperatures in household refrigerators and retail display units frequently exceed the recommended 5°C and often reach or surpass 8°C (14). The selected strain had previously demonstrated the ability to internalize into plant tissues (15), indicating tolerance of plant-associated environments. We therefore considered it a suitable comparative model to investigate whether a plant-associated yet host-adapted serovar can deploy genetic determinants that support persistence on fresh produce matrices. To explore this, we generated a bar-coded transposon insertion sequencing (TIS) library pool as well as an ordered library of individual insertion mutants (IMs) in this *S. enterica* serovar Choleraesuis strain, 11-Ga-2015 (SCH).

Overall, this study examines genome-wide fitness mechanisms in a serovar traditionally considered host-adapted to pigs, a group rarely implicated in foodborne transmission yet often associated with invasive and severe disease outcomes when infections occur (16). By characterizing persistence mechanisms in this serovar under shelf-life conditions, this work expands current knowledge of how adaptation and persistence strategies may vary among environmental conditions and adaptation strategies beyond the most commonly studied serovars. These findings support the identification of genetic determinants relevant to survival on fresh produce, specifically alfalfa sprouts, and contribute to the development of precision food safety interventions designed to address the diversity of *Salmonella* serovars capable of contaminating fresh produce systems.

## MATERIALS AND METHODS

### Bar-coded transposon insertion sequencing library construction in *S.* Choleraesuis

*S. enterica* serovar Choleraesuis strain 11-Ga-2015 (SCH) (17) (accession no. JADWDF000000000) was used to construct a bar-coded TIS library. This strain was originally isolated from the pulp of a Galia melon (15) from a German supermarket in 2015 and had been preserved at −80°C in Luria–Bertani broth (LB; Carl Roth GmbH & Co. KG, Karlsruhe, Germany) with 20% glycerol (Th. Geyer GmbH & Co. KG, Renningen, Germany).

The construction of the bar-coded Tn*5*-based mutant library was performed as previously described (18, 19), with minor modifications. Briefly, an N_21_ random barcode flanked by Illumina primer sequences was inserted into the EZ-Tn*5* transposon via a two-step PCR, using primers Tn*5*PCR1L and Tn5PCR1R in the first step and the following cycling conditions: 98°C 90 s; 35× (98°C 10 s, 62°C 20 s, and 72°C 115 s); final extension 72°C 3 min; hold 8°C. The second PCR step was performed using primers 5P-Tn5PCR2L and P-Tn5PCR2R with similar cycling conditions: 98°C 90 s; 30× (98°C 10 s, 61°C 20 s, and 72°C 115 s); final extension 72°C 3 min; hold 8°C. All primer sequences are shown in Table S1. The purified construct was subsequently randomly introduced into the SCH genome using EZ Tn*5* transposase (Epicenter Biotechnologies, Madison, WI, USA), as per the manufacturer’s recommendations. Transformed cells were harvested from LB^kan60^ (60 μg/mL kanamycin, Carl Roth) agar after an overnight culture at 37°C.

The methods for mapping the bar-coded transposons to specific locations in the genome have previously been described (18). For the bar-coded SCH TIS library, extraction of the genomic DNA was performed using the GenElute bacterial genomic DNA kit (Sigma-Aldrich, St. Louis, MO, USA), followed by a standard 2 h linear amplification reaction with a temperature ramp to 37°C using a random N_10_ primer (KlenowN_10_) and exo- Klenow enzyme (New England Biolabs [NEB], Ipswich, MA, USA). Deactivation was performed at 75°C for 20 min, and products were purified using the QIAquick PCR Product Purification kit (QIAGEN, Maryland, USA). The region that includes the N_21_ barcode and the genomic DNA adjacent to the transposon was amplified using a nested PCR protocol that included Illumina primer sequences. Primer pair Klenow_PCR1SCHL/Klenow_PCR1SCHR was used in the first step, whereas Klenow_PCR2SCHL, including the Illumina P5 adapter, was paired with outward-facing indexed primers that included the Illumina Read2 and P7 adapter in the second step. After QIAquick purification of the resulting amplicons, paired-end 150-base reads were obtained, and the relevant 71-base region was mapped to the *de novo* assembled SCH genome using CLC Genomics Workbench (v24; QIAGEN, Venlo, Netherlands). Approximately 33,000 individual barcodes were mapped to the SCH genome.

### Establishment of the ordered SCH transposon library

To make an ordered SCH transposon library, 9,595 individual SCH Tn mutant clones were toothpicked from LB^kan60^ agar plates during the generation of the TIS library prior to pooling. They were grown separately in LB^kan60^ in a total of 101 96-well plates (“source plates”). All mutants from each plate were pooled (resulting in 101 “plate pools,” each consisting of 95 mutants), and all mutants from the same wells across all plates were also pooled (resulting in 95 “well pools,” each with 101 mutants). The 101 plate pools and the 95 well pools were then prepared for sequencing by proteinase K digestion and PCR amplification using primers PCRSCHoTnL and PCRSCHoTnR to create Illumina sequencing libraries. Illumina sequencing was used to identify each N_21_ barcode. Analysis of barcode occurrence in the plate pools and well pools revealed the exact plate/well coordinates of each bar-coded mutant in the source plates, while prior mapping had revealed the location of each bar-coded transposon insertion in the SCH genome.

For the final selection, each mutant that was the sole representative of a genetic feature of SCH was included. When multiple mutants were available for a given gene, two clones per gene were selected. Priority was given to clones with transposon insertions on opposite strands of the gene, preferably near the N-terminus, while maintaining a minimum distance of 10 bases from gene boundaries.

### Comparative growth analysis

Population dynamics of the bar-coded SCH TIS library on alfalfa sprouts were analyzed in five replicates and statistically compared to those of the wild-type (WT) strain 11-Galia-2015. Inoculation and sampling were performed at 8°C as described for the library screenings below. An ANOVA test was used for statistical analysis, with adjusted *P* values of <0.05 considered statistically significant.

### Library screening on alfalfa sprouts during cold storage

The methods for the TIS library screen on food matrices were previously described (20, 21). In brief, 300 μL of library stocks was thawed and propagated in 30 mL of LB^kan60^ broth at 37°C and 200 rpm until OD_600_ reached 1.0. After centrifugation of the 10-fold diluted culture (4,500 rcf, 5 min), the supernatant was discarded, and the inoculum was prepared by resuspending the cell pellet in 5 mL of phosphate-buffered saline (PBS, Carl Roth).

Alfalfa sprouts (*Medicago sativa* L.) from the same producer were purchased from local supermarkets. According to the product information, sprouts were stored under refrigerated conditions after harvest. The cold chain was maintained until experimentation, except for brief interruptions during sample weighing. To quantify the background microbiota of the uninoculated sprouts, mesophilic aerobic bacteria (MABs) were enumerated according to EN ISO 4833-2:2013, and *Enterobacteriaceae* were quantified following EN ISO 21528-2:2017 (anaerobic incubation conditions: O_2_ < 0.1%, CO_2_ 7.0%–15.0%) at each sampling time point. The absence of *Salmonella* spp. in sprout samples was verified by qualitative microbiological analysis, in accordance with EN ISO 6579-1:2017.

For inoculation, 10 g of alfalfa sprouts was aseptically transferred into sterile filter Stomacher bags (0.4 L; Avantor VWR International GmbH, Darmstadt, Germany). Each sprout sample was mixed with 5 mL of the inoculum, while control samples received only 5 mL of PBS. The final inoculum concentration was approximately 8 log_10_ CFU/mL with a final concentration of 7 log_10_ CFU/g sprouts to maintain library complexity. All samples were incubated at 8°C for 5 days.

Sampling of the mutant libraries was performed directly from the inoculum as well as at 1 h (d_1_), 48 h (d_3_), and 96 h (d_5_) post-inoculation. At these time points, alfalfa sprouts and negative control samples were transferred to 90 mL of LB^kan60^ in Erlenmeyer flasks and incubated at 37°C with shaking at 200 rpm for 30 min to detach bacteria from the matrix. *Salmonella* populations were subsequently quantified by plating serial dilutions on BPLS agar (Merck KGaA, Germany), which was incubated at 37°C for 24 h. Population dynamics of the bar-coded SCH TIS library on sprouts and in PBS were statistically compared using ANOVA, with *P*_adj_ values of <0.05 considered significant.

In parallel, samples in 90 mL LB^kan60^ were filtered through the Stomacher bags and centrifuged (4.500 rcf, 5 min) to eliminate residual plant material in the medium, and the pellet was resuspended in 90 mL fresh LB^kan60^. Sprout samples and negative controls were then further incubated for 7.5 h at 37°C and 200 rpm. From this, 1 mL aliquots were stored at −80°C in broth without glycerol for subsequent genomic DNA extraction and sequencing. Each experiment was performed in five biological replicates.

For sequencing library preparation, 40 μL aliquots were washed three times with sterile ultrapure water and pelleted. Washed cells (15 μL) were subjected to a two-step lysis protocol. In the first step, cells were incubated with 1.6 μL lysozyme (10 mg/mL; AppliChem GmbH, Darmstadt, Germany) in 8 μL lysis buffer consisting of 10 mM Tris (pH 8.0), 1 mM EDTA (Carl Roth), and 0.1% Triton X-100 (Carl Roth) for 5 min at 20°C, followed by enzyme inactivation at 98°C for 5 min and cooling to 4°C. In the second step, 1 μL proteinase K (100 mg/mL, Sigma-Aldrich) was added in 5 μL lysis buffer and incubated for 2 h at 55°C with subsequent proteinase K inactivation at 95°C for 10 min and immediate cooling. After centrifugation for 1 min at 14,100 rcf, 5 μL of the lysate was used as template for PCR amplification with indexing primers annealing to sequences flanking the transposon barcode (Illumina-bar-coded primers N and S, 0.2 μM each; see Table S1). PCR amplification was carried out using Q5 Hot Start High-Fidelity 2× Master Mix (NEB) at a final 1× concentration. The cycling protocol consisted of an initial denaturation at 98°C for 2 min, followed by 10 cycles of 98°C for 10 s, 65°C for 10 s, and 72°C for 20 s. Additional 20 cycles were then performed with denaturation at 98°C for 10 s and extension at 72°C for 20 s. A final elongation step was conducted at 72°C for 2 min, after which reactions were held at 20°C. Amplified products were pooled in equimolar volumes and purified using the QIAquick PCR Purification Kit (QIAGEN) according to the manufacturer’s protocol. Sequencing libraries were analyzed by dual-indexed paired-end sequencing (100–150 bp reads) on an Illumina NovaSeq 6000 platform. On average, 1,057,353 mapped barcodes (minimum of 736,054 and maximum of 1,417,144 mapped barcodes) were sequenced per sample.

### Bioinformatic analysis

Raw reads from different samples were demultiplexed by Illumina index and analyzed with custom Python scripts to identify and quantify barcodes flanked by invariant sequences. Barcode counts were aggregated per disrupted gene, and changes in mutant abundance across time points and between “matrix”and “no matrix” samples were statistically assessed using DESeq2 (22), with log_2_ fold changes (fc) reported (Table S2).

Mutations were considered to have a significant fitness effect for SCH on alfalfa sprouts if, at a given sampling time point, they showed (i) a log_2_ fc of >∣1.0∣ with a *P*_adj_ value of ≤0.01 when comparing the populations on sprouts vs PBS (*no matrix* sample) and (ii) the same significance threshold (log_2_ fc >∣1.0∣, adjusted *P*_adj_ ≤0.01) when comparing the inoculum to the sprout sample at the same sampling time point. This dual-filtering approach ensured that only the genes most relevant to the interaction between SCH and sprouts were identified.

To prepare gene lists for input into KEGG (23) and Gene Ontology (GO) (24-26) enrichment analyses, the adjusted *P* value threshold for significance was relaxed from ≤0.01 to <0.05. GO enrichment was performed using the TopGO package (27), while KEGG pathway analysis was conducted with the enrichKEGG function from the ClusterProfiler package (28) in R (v4.4.1). KEGG pathways with *q* values ≤0.01 and GO terms with classic Fisher *P* values ≤0.01 were considered enriched.

### Competition assays

The phenotypes of candidate genes identified in the transposon analysis were verified using selected individual IMs from the ordered SCH transposon library in direct competition with the WT strain. For some of these candidate genes, two mutants were available—one harboring the transposon insertion in the sense direction and the other harboring the transposon insertion in the antisense direction—facilitating the identification of orientation-dependent effects. The insertion mutants analyzed in this study are listed in Table 3.

For competition assays, a single colony of the IMs and the WT strain were cultured overnight at 37°C with shaking at 200 rpm in 5 mL LB^kan80^ (80 μg/mL) and LB, respectively. From the overnight cultures, 300 μL was cultured in 30 mL LB^kan80^ (80 μg/mL) and LB, respectively, under the same conditions until an OD_600_ of 0.9 was reached. IM and WT pairs were then mixed at a 1:1 ratio and diluted 1:10 in PBS, and 1 mL of this IM-WT mix was centrifuged. Pellets were resuspended in 5 mL PBS, and the centrifugation and resuspension steps were repeated once to wash the cells. Subsequently, 5 mL was used to inoculate 10 g sprout samples and corresponding no matrix control bags containing only the inoculum mixture. To control nutrient-dependent effects, parallel competitions were resuspended in LB medium instead of PBS. The final inoculum concentration on the samples was approximately 7.0 log_10_ CFU/mL or g sprouts. All samples were incubated at 8°C. The specific incubation period for each candidate gene was selected based on the time point showing the strongest fitness effect in the transposon screen. To assess changes in population ratios, samples were subsequently plated on both BPLS and BPLS^kan80^ agar and incubated at 37°C for 24 h. WT colony counts were calculated by deducting the CFUs observed on BPLS^kan80^ from those on BPLS.

The phenotype of the candidate genes was confirmed by comparing colony count changes of the respective sense and antisense IM and the WT strain, and calculating the corresponding competition indices [competition index (CI) = (mutant / wild type) × time point / (mutant / wild type) × inoculum] for each experiment. A negative effect was confirmed if the average CI of all the replicates plus the standard deviation (Stdev) was <1. If the average CI minus the standard deviation was >1, a positive fitness effect was confirmed. Competition assays were performed in at least three independent experiments.

## RESULTS

### Generation of a bar-coded *S.* Choleraesuis TIS library and an ordered mutant collection

Our electroporations generated an S. Choleraesuis transposon mutant library consisting of 35,432 independent mutants with mapped Tn5 insertions, 34,811 of which were mapped to the genome and 621 to the plasmid. The mapped insertions disrupted 3,684 of 4,624 annotated chromosomal genes (80%) and 53 of 57 annotated plasmid genes (93%). Many of the undisrupted genes are probably essential for SCH under laboratory conditions. The library also contained insertions in 1,579 intergenic regions (IRs).

Additionally, we generated an ordered SCH transposon mutant collection consisting of 5,304 individual clones. This collection represents 2,838 of the 4,682 annotated SCH genes, corresponding to 61% coverage of the coding genome. For the 1,659 genes with multiple available mutants, two clones were included per gene, with priority given to insertions located on opposite strands and positioned near the C- or N-terminus while avoiding the first or last 10 bases of the gene. In addition to coding regions, the library also includes mutants affecting 807 of the 3,807 annotated intergenic regions. Overall, this curated library provides a broad representation across the SCH genome, enabling systematic functional analyses of both genes and intergenic elements.

### *S.* Choleraesuis and native microbiota dynamics on alfalfa sprouts

Statistical analysis of the bar-coded SCH TIS library’s dynamics on alfalfa sprouts revealed no statistically significant differences compared to the population dynamics of its WT (*P*_adj_ > 0.05, one-way ANOVA) for any of the sampled time points (Fig. S1).

**FIG 1:**
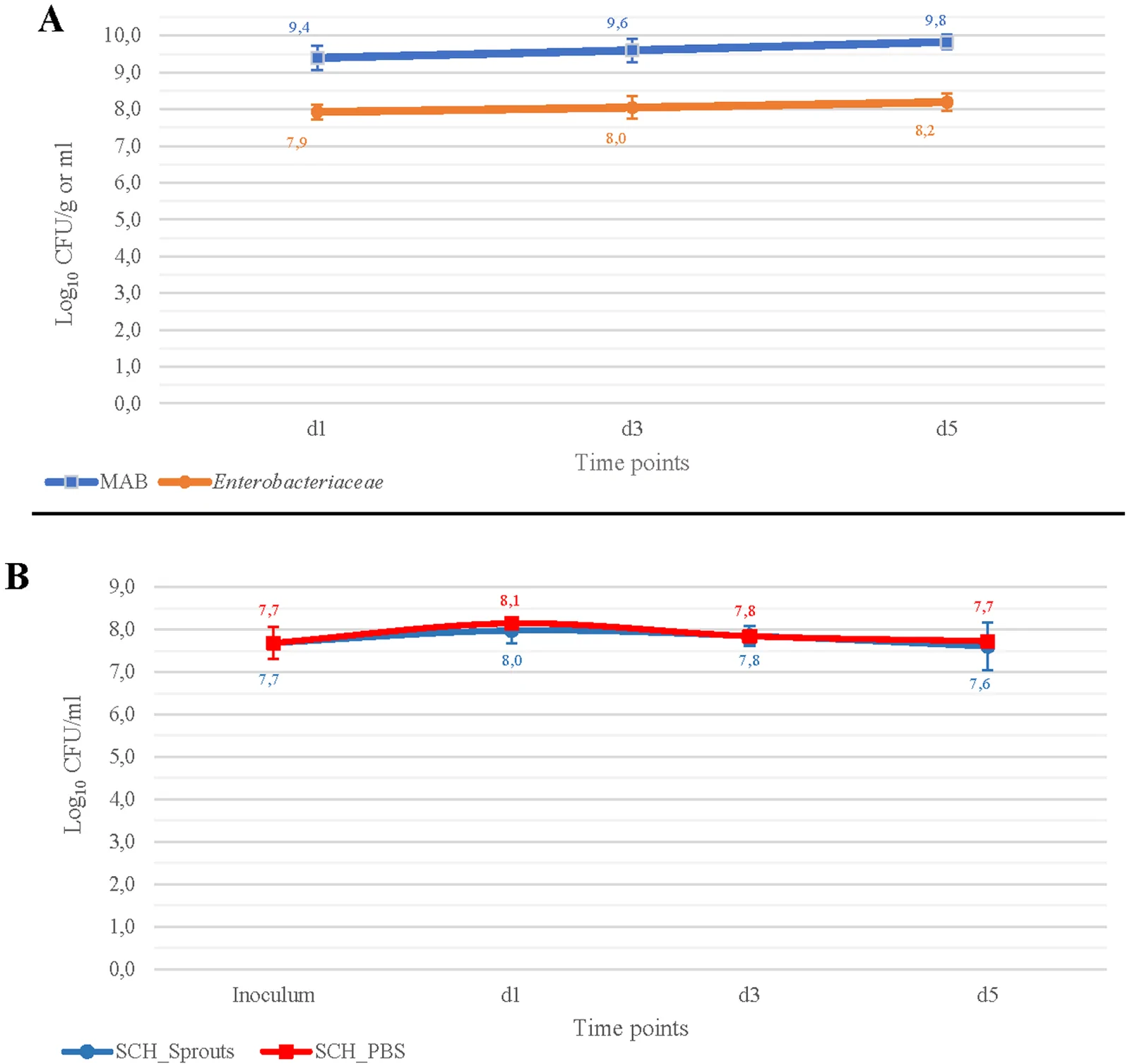
Population dynamics on alfalfa sprouts at 8°C. (A) Dynamics of the background microbiota, represented by mesophilic aerobic bacteria (MABs) and *Enterobacteriaceae*, at 1 h (d_1_), 48 h (d_3_), and 96 h (d_5_) post-inoculation. (B) Quantitative assessment of the *S.* Choleraesuis (SCH) bar-coded TIS library in the inoculum and at the same sampling time points on alfalfa sprouts (SCH_Sprouts) and in PBS (SCH_PBS). There was no significant (*P* > 0.05) difference between the SCH library populations on alfalfa sprouts and in PBS during cold storage. Data represent averages from five biological replicates, with error bars indicating standard deviations. SCH, bar-coded TIS library in *S.* Choleraesuis 11-Ga-2015.

Uninoculated alfalfa sprout samples were prepared and incubated under the same conditions as the screening samples to monitor the initial background microbiota and its dynamics in the absence of *Salmonella*. There was a high microbial load on alfalfa sprouts with initial MAB counts reaching 9.4 log_10_ CFU/g and *Enterobacteriaceae* populations approaching 8.0 log_10_ CFU/g. The bacterial load remained stable during the full incubation period (96 h) at 8°C (Fig. 1A) with low variability among samples. No macroscopic changes were observed in the appearance of the inoculated alfalfa sprouts during the incubation period, consistent with commercially available sprouts.

The dynamics of the SCH library populations on alfalfa sprouts and in PBS at 8°C were not significantly different (*P*_adj_ > 0.05) and remained largely stable throughout the incubation period (Fig. 1B). Importantly, no net population growth was observed under these conditions, indicating that the experimental setup reflects a non-proliferative, persistence-like state.

### Identification of *S.* Choleraesuis genes with a role during abusive cold storage on alfalfa sprouts

**FIG 2:**
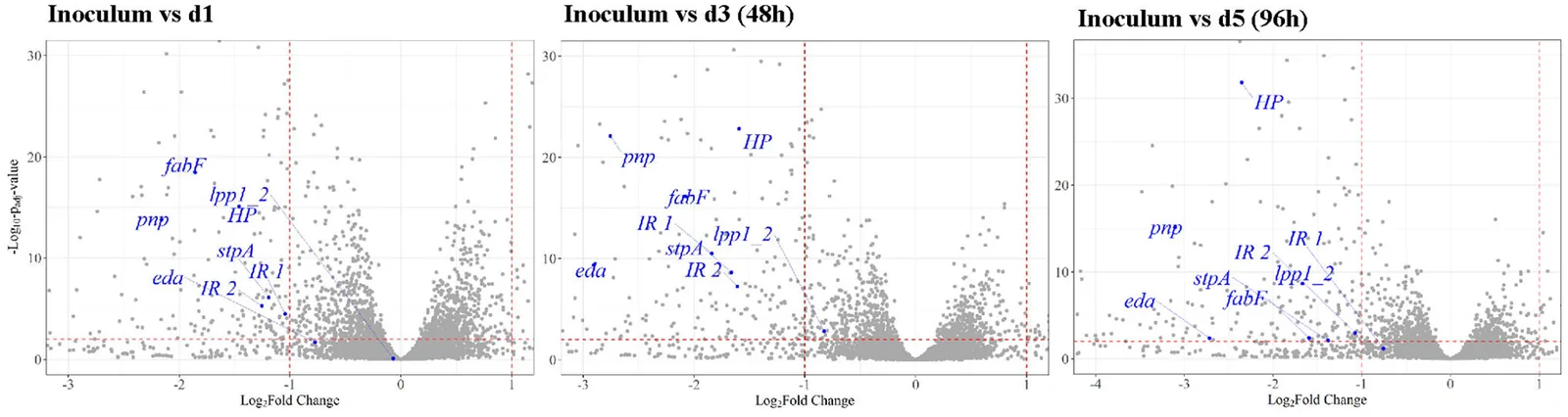
Changes in mutant abundances of S. Choleraesuis 11-Ga-2015 on alfalfa sprouts at 8°C. Volcano plots illustrate genome-wide fitness effects, with the log_2_ fold change in relative mutant abundance on the *X*-axis and the adjusted *P* values on the *Y*-axis. Comparisons are shown between the inoculum and samples collected 1 h post-inoculation (d_1_), 48 h post-inoculation (d_3_), and 96 h post-inoculation (d_5_). Each dot represents an individual insertion mutant, and mutants that fulfilled the dual significance criterion are labeled in blue. Red dotted lines indicate the thresholds for significance in each comparison.

Using our stringent two-pronged threshold for significance, we found that only eight SCH genetic elements showed a statistically significant fitness effect during a 5-day cold storage on alfalfa sprouts when analyzed with the SCH random transposon library: six coding genes (*eda*, *fabF*, *lpp1_2*, *pnp*, *stpA*, *SCHChr_03621*; Table 1) and two IRs located between *deaD* and *nlpI* as well as between *pipB* and *sigE*. Further analyses of the genes delimiting the IRs showed that, while *nlpI* mutants remained mainly stable during the interaction with alfalfa sprouts, *deaD* mutants were negatively selected with stronger reductions in the sprout samples than in the negative controls but did not meet the statistical threshold for the sprouts vs *no matrix* comparison. While the insertion within the *pipB-sigE* IR was deleterious, disruption of the two flanking genes caused no fitness effects. Mutant dynamics during the screening period are represented in Fig. 2.

KEGG enrichment analysis of genes affecting the fitness of SCH during cold storage on alfalfa sprouts was performed to provide an overview of relevant pathways for the interaction of *S.* Choleraesuis with this matrix. “Flagellar assembly” was significantly enriched (*q* ≤ 0.01) together with three associated GO terms (*P* ≤ 0.01), as summarized in Table 2. Analysis of the transposon data revealed that all selected genes associated with flagellar assembly exhibited an overall positive selection, which was particularly pronounced during the first hour of interaction but shifted to a mild negative effect between 1 h post-inoculation and the final time point (96 h).

Additionally, GO terms “nitrogen fixation” (GO:0009399, *glnGA*) and “enterobacterial common antigen biosynthetic process” (GO:0009246; *wecBE*, *wzyE*, *rffM*) were significantly enriched.

### Competition assays confirm genetic determinants

Insertion mutants in *eda*, *fabF*, *lpp1_2*, *mnmG*, and *stpA* were selected from the available mutants in the ordered SCH library collection for direct competition against the WT (Table 3). Among these, *mnmG* (also named *gidA*) did not meet the dual requirement for fitness effect significance but was included due to the strong reduction in mutant abundance and previous TIS data, suggesting a recurring pattern on this food matrix (21).

For *stpA* and *mnmG*, two individual IMs were available in both the sense and antisense orientations. For the remaining three genes, only one mutant was present: *fabF* with the transposon inserted in the sense orientation, and *eda* and *lpp1_2* with insertions in the antisense orientation. The sampling time point selected for the competition assays corresponded to that showing the strongest fitness effect in the transposon screen. Assays were performed on alfalfa sprouts and in nutrient-rich LB broth as a control to distinguish fitness effects associated with general nutrient availability from those specific to the alfalfa sprout environment. Competition assays were also run in PBS control conditions. The results are summarized in Table 3.

A negative fitness effect was confirmed when the CI plus the Stdev was less than 1 for both IMs, while a positive fitness effect was defined by a CI – Stdev >1 for both mutants. For genes with only one available IM, meeting the described threshold in that single mutant was considered sufficient to validate the phenotype observed in the transposon assay.

## DISCUSSION

This study investigated fitness determinants of the plant-derived *S.* Choleraesuis strain 11-Ga-2015 on alfalfa sprouts during abusive cold storage at 8°C, reflecting temperature conditions frequently encountered during retail display and domestic storage, where recommended refrigeration limits are often exceeded (14, 29). To approximate real-world conditions, experiments were performed on commercially available alfalfa sprouts without microbiota reduction. This design therefore allowed the identification of fitness determinants under conditions that closely resemble real-world storage environments, where native microbiota and fluctuating temperature conditions influence pathogen persistence.

Quantitative analysis showed that overall *S.* Choleraesuis TIS library populations increased slightly within the first hour of interaction with sprouts but subsequently declined over the remaining time of storage. Although data on *S. enterica* growth dynamics on sprouts under cold conditions remain limited, our previous analyses of *S.* Typhimurium and *S.* Enteritidis on alfalfa sprouts under identical experimental conditions (21) showed no initial growth phase.

The consistently high background microbiota observed on alfalfa sprouts, as reported here and in previous studies (4, 21, 30), together with their association with multiple foodborne outbreaks (3), most commonly involving *Salmonella* and enterohemorrhagic *E. coli*, highlights the potential of sprouts to serve as a favorable niche for microbial persistence and growth. This aligns with the outcomes of our transposon screens of *S. enterica* libraries on alfalfa sprouts, where only a limited number of genetic elements showed significant fitness effects, which may be associated with the relatively permissive conditions of alfalfa sprouts at 8°C. Specifically, mutations in six *S.* Choleraesuis genes and two IRs conferred a fitness disadvantage, whereas 13 genes and 5 elements were identified in *S.* Enteritidis and *S.* Typhimurium, respectively, in our previous study (21).

Mutants carrying insertions in genes associated with carbohydrate metabolism (*eda*), RNA degradation (*pnp*), lipid metabolism (*fabF*), cell wall structure and interaction mechanisms (*lpp1_2*), DNA structuring (*stpA*), and an hypothetical protein (*SCHChr_03621*) exhibited significant fitness effects under the tested conditions. Competition experiments in *eda*, *fabF*, *lpp1_2*, *stpA*, and *mnmG* SCH mutants confirmed negative fitness effects for *eda*, *fabF*, and *mnmG* specifically in alfalfa sprouts but not in nutrient media or PBS. These findings underscore the importance of carbohydrate and lipid metabolism, as well as RNA degradation and translational control, as key processes supporting *S.* Choleraesuis fitness in this ecological niche.

**FIG 3:**
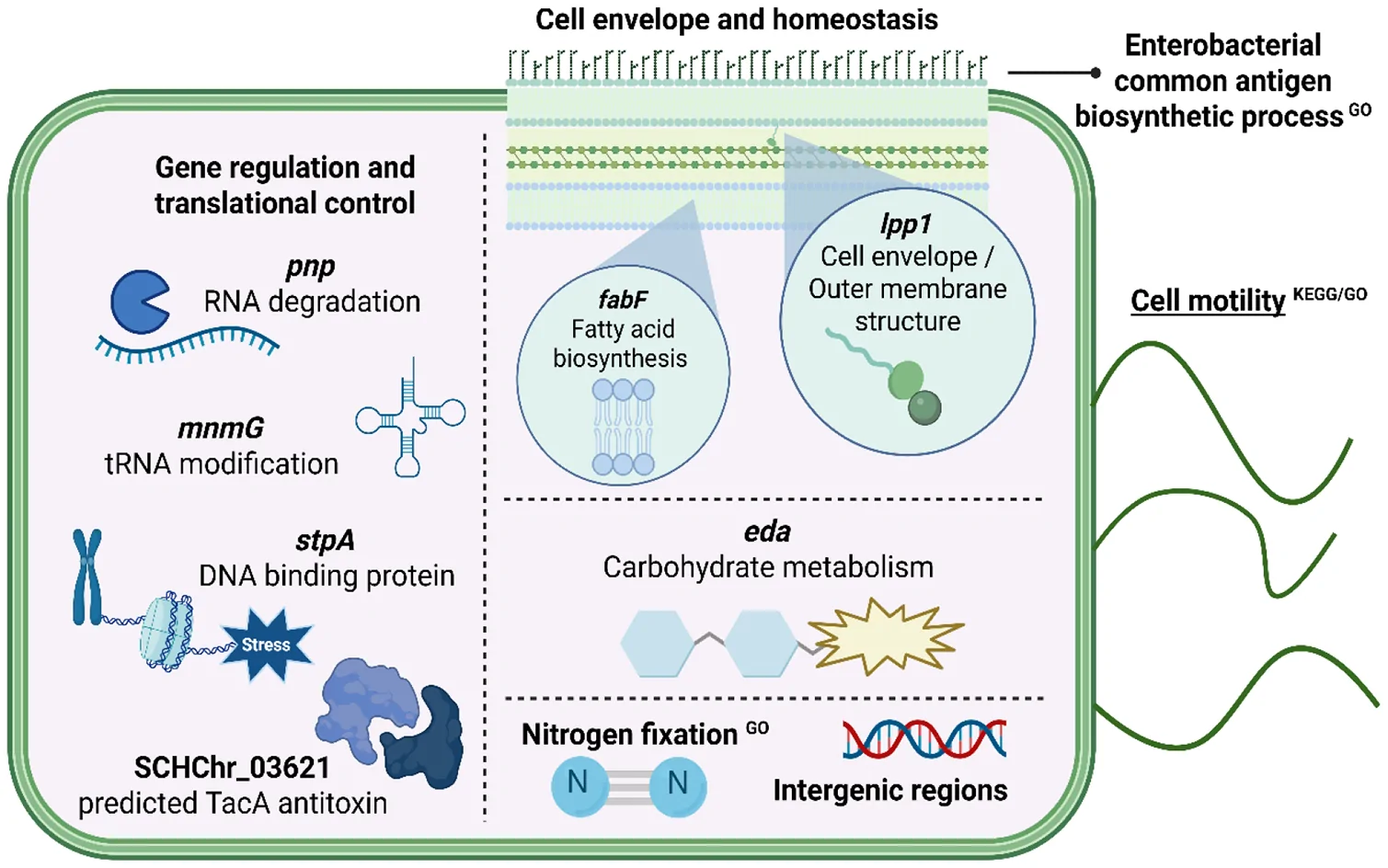
Overview of genetic element contributing to *S.* Choleraesuis fitness on alfalfa sprouts. Genetic elements as well as enriched KEGG pathways and GO terms identified in the transposon screen were grouped according to their cellular functions. Underlined functions were positively selected, in contrast to not underlined functions, which showed a negative selection. Created in BioRender. Beck, M. (https://BioRender.com/5ulmitz).

The identified genes do not belong to a single operon or linear pathway, but their predicted functions can be organized into distinct, biologically coherent modules, as summarized in Fig. 3. One major module, including *pnp*, *mnmG*, *stpA*, and the predicted TacA family antitoxin SCHChr_03621, corresponds to gene regulation and translational control. The negative selection of these determinants suggests that RNA turnover, tRNA modification, nucleoid-associated regulation, and persistence-related stress responses contribute to the fitness of SCH under the tested conditions. A second module corresponds to functions involved in cell envelope structure and adaptation to environmental challenges, including *fabF* and *lpp1_2*, as well as the enterobacterial common antigen biosynthetic process.

The pronounced fitness defect observed in *eda* mutants highlights the critical role of the carbohydrate metabolism—and specifically the Entner–Doudoroff (ED) pathway—in SCH fitness on alfalfa sprouts. The *eda* gene encodes KDPG (2-keto-3-deoxy-6-phosphogluconate) aldolase, a central enzyme of the ED pathway that operates in parallel with the classical Embden–Meyerhof–Parnas (EMP) glycolysis. Although the ED pathway yields only half as much ATP as the EMP pathway, recent work has revealed that it confers a distinct selective advantage under dynamically changing nutrient conditions (31). Specifically, the ED pathway enables rapid metabolic acceleration upon carbon and nitrogen upshifts. This capacity for rapid flux adaptation likely provides a competitive advantage in fluctuating microenvironments such as plant surfaces, where nutrient availability is spatially heterogeneous and temporally intermittent. Consistent with this, the ED pathway was shown to be required in the challenging environment inherent to various host infection processes, promoting *Salmonella* proliferation within macrophages and epithelial cells (32, 33).

The relevance of this pathway is further underscored by the fitness effects of *S. enterica eda* mutants on various foods of plant origin. On melons, *eda* was critical for both *S.* Typhimurium and *S.* Enteritidis at room temperature, whereas its contribution was marginal at 8°C (20). The phenotype observed at ambient temperature likely reflects a rapid upshift in glucose availability following melon processing, as cutting enhances nutrient release. Under conditions that support active growth, the ED pathway may facilitate rapid carbon flux and thereby contribute to bacterial fitness. On sprouts, *S.* Typhimurium *eda* mutants also exhibited a negative fitness effect at 8°C (21). Collectively, these observations highlight the importance for SCH and, more broadly, for *S. enterica*, of efficiently managing energy and carbon resources in various fresh produce matrices, such as sprouts and melon.

The effects observed for *pnp* mutants highlight the role of RNA degradation in bacterial adaptation to the plant-associated environment by contributing to resource recycling and optimization. The *pnp* gene encodes the enzyme polynucleotide phosphorylase (PNPase), a highly conserved component of the RNA degradosome with established roles in mRNA turnover, tRNA processing, and sRNA-mediated regulation (34). Compared to SCH, *pnp* mutants of *S.* Typhimurium or *S.* Enteritidis exhibited relatively mild fitness defects on sprouts (21). Nevertheless, the importance of *pnp* for environmental resilience is evident across other produce-associated contexts: it contributes to the survival of *S.* Typhimurium on ready-to-eat muskmelons (20) and onions (21), and transposon analyses similarly implicate it for *S.* Enteritidis and *S.* Newport fitness on muskmelons (20). Disruption of *pnp* also reduces fitness on low-moisture foods, including almonds (*S.* Enteritidis) and pistachios (*S.* Typhimurium) (35). Competition assays in nutrient-rich conditions (LB) provided an important control: while *S.* Typhimurium *pnp* mutants showed no fitness defects at 22°C (20), they exhibited a significant disadvantage at 8°C on onions (21) and in this study, consistent with the established role of PNPase in the bacterial cold-shock response. Although *S. enterica pnp* mutants display milder cold sensitivity compared to other bacteria (34), their reduced fitness at low temperatures underscores the enzyme’s contribution to maintaining cellular function under cold stress.

We observed a significant fitness effect in SCH *fabF* mutants on alfalfa sprouts. FabF (β-ketoacyl-acyl carrier protein synthase II) catalyzes fatty acid (FA) elongation, particularly contributing to the synthesis of long-chain and unsaturated FAs (36). This activity is critical for maintaining membrane fluidity, permeability, and overall cell envelope integrity. Although SCH encodes other β-ketoacyl-ACP synthases, including FabB, these enzymes cannot fully compensate *fabF* mutations: in addition to catalyzing FA elongation, FabF also modulates chain length in response to temperature changes and is less sensitive to inhibitors like cerulenin (36). This further highlights the specialized role of FabF in maintaining membrane integrity and adapting lipid composition under temperature-dependent stress conditions.

The observed fitness defect in SCH *fabF* mutants was specific to alfalfa sprouts and absent in LB or PBS, indicating that the contribution of FabF is food matrix dependent and particularly critical for SCH survival on alfalfa sprouts under abusive cold storage. Low temperatures reduce membrane fluidity, and bacteria adapt by increasing the proportion of unsaturated fatty acids to maintain membrane homeostasis (37). Consistent with this role, *fabF* mutants in *E. coli* (38), *Clostridium acetobutylicum* (39), and deep-sea bacteria (40) exhibit cold-sensitive phenotypes, highlighting the conserved function of FabF in cold adaptation.

Membrane lipid composition changes not only in response to cold stress but also as a result of desiccation. Accordingly, *fabF* mutants exhibited a negative fitness effect under desiccation stress in *S.* Typhimurium on pistachios stored at 25°C (41), and in *E. coli*, desiccation triggers adjustments in FA composition to preserve membrane fluidity (42). Together, these observations underscore the central role of FA metabolism in maintaining membrane integrity.

The observed fitness effects of *fabF* and *lpp* mutants are likely interconnected. In *Salmonella*, the outer membrane murein lipoprotein Lpp is encoded by two genes, *lpp1* and *lpp2*, and disruption of either results in reduced virulence strains (43). Proper membrane function depends on maintaining bilayer integrity, which is supported in part by the regulation of lipid A biosynthesis (36). Deficiencies in FA biosynthesis mediated by *fabF* can indirectly perturb the metabolism of envelope-associated polysaccharides, including lipopolysaccharides (LPS) and the enterobacterial common antigen because its products supply the phospholipids for the inner leaflet of the outer membrane (36, 44). LPS, including genes involved in O-antigen biosynthesis, have previously been shown to play an important role in the interaction of *S. enterica* with sprouts (11, 21) as well as with other foods of plant origin, including low-moisture foods, onions, and melons (20, 21, 35, 41).

This effect may also relate to the association of Lpp with the Tol-Pal system, a five-component transmembrane complex whose disruption compromises outer membrane integrity, alters LPS composition, and increases susceptibility to multiple stressors, including antimicrobial compounds such as polymyxins (45). Genome analysis of SCH showed that *lpp1* is represented by two CDS: *lpp1_1* and *lpp1_2*. Only mutants in *lpp1_2* were present in the SCH bar-coded transposon library, and these exhibited a significant negative fitness effect on sprouts, whereas mutants in *lpp2*—located between *lpp1_2* and *lpp1_1* in the antisense orientation—did not.

Competition assays between the *lpp1_2* SCH insertion mutant and the WT revealed no fitness disadvantage for the mutant under any of the tested conditions (sprouts, LB, and PBS). This may indicate functional redundancy between *lpp1_1* and *lpp1_2*, warranting further investigation through the construction of a *lpp1_1/2* double mutant. However, the apparent signal might also represent a bioinformatics artifact rather than a genuine biological phenomenon.

SCH mutants in *mnmG* (also referred to as *gidA*) exhibited pronounced reductions in fitness on alfalfa sprouts and, in previous studies, for *S.* Typhimurium on onions and sprouts (21). Although these effects fell just below the threshold of statistical significance relative to control samples, *mnmG* emerged as a gene of interest for the competition experiments, which confirmed a matrix-specific fitness defect for *mnmG* mutants on alfalfa sprouts.

MnmG (glucose-inhibited division protein A) is a highly conserved tRNA modification enzyme that, together with MnmE, catalyzes the addition of a 5-carboxylmethylaminomethyl group to the wobble uridine of tRNAs. This modification is critical for efficient and accurate translation, influencing global protein synthesis and regulation of diverse cellular processes. In *S. enterica*, *mnmG* also modulates the expression of virulence-associated genes, regulating pathogenesis *in vivo* and *in vitro* infection models, and influences growth, stress tolerance, and biofilm formation (46, 47).

Although a general fitness defect might be anticipated for *mnmG* mutants, the phenotype was particularly pronounced under the specific stress conditions present on alfalfa sprouts. This environment likely imposes a combination of selective pressures, such as variable nutrient availability, plant-derived oxidative and antimicrobial compounds, and challenges associated with colonization and abusive cold storage conditions, which together may contribute to the critical role of *mnmG* in SCH fitness in this ecological niche.

The *SCHChr_03621* mutant showed a significant fitness defect in SCH in the transposon assay. Sequence comparison using the Uniprot database (48) and blast+ (49) predicts that *SCHChr_03621* encodes the antitoxin component of a TacA/TacT family type II toxin–antitoxin (TA) system. Non-secreted type II TA system toxins in *Salmonella* are induced inside host macrophages, leading to growth arrest and the formation of metabolically active, non-growing persister populations (50, 51). TacT induces growth arrest in *S.* Typhimurium by stalling the ribosome, a process that is reversible by the activity of the antitoxin TacA (encoded by *pth*) (52). We therefore hypothesize that conditions on alfalfa sprouts may similarly trigger TacT expression, rendering the loss of the antitoxin *tacA* detrimental to bacterial fitness.

Competition experiments between SCH *stpA* mutants and the WT revealed an orientation-dependent negative fitness effect in the antisense orientation of *stpA*. Located upstream of *stpA* is *SCHChr_02838*, a rhodanese-related sulfurtransferase encoded in a separate operon. Insertion mutants in this locus did not display significant fitness defects in the transposon assays on alfalfa sprouts, indicating that the fitness defect is unrelated to *SCHChr_02838*. Examination of the transposon insertion sites of both *stpA* insertion mutants revealed that the sense-oriented insertion is positioned near the 3′ end of the gene, whereas the antisense insertion lies centrally within the coding sequence. This positional difference could indicate that the sense-oriented insertion may not disrupt essential functional domains of StpA, suggesting that the observed fitness defect would correspond to the disruption of *stpA* itself.

Together with previous transposon-based screens in *Salmonella* on sprouts, melons, onions, and low-moisture foods, our findings support the view that produce-associated fitness is governed by recurring functional themes, including carbon metabolism, RNA metabolism, envelope integrity, LPS/O-antigen-related traits, and motility-associated functions (11, 20, 21, 41, 53). Although common functional themes are retained, the underlying genetic determinants can vary among serovars and across food matrices, while certain conserved contributors, such as *pnp*, may still be identified.

Flagellar assembly was significantly enriched in both KEGG and GO analyses, with mutant abundances of six selected genes showing an initial negative selection that shifted to positive over time. This dynamic has been observed in previous studies on alfalfa sprouts (11, 21) and melons (20). We hypothesize that the loss of flagella may initially disadvantage individuals by reducing their ability to access nutrient-rich microenvironments and avoid localized stressors on plant surfaces. However, the plant immune system can detect bacterial flagella, and mutants may gain a competitive advantage during colonization, as was shown in *Listeria monocytogenes* (54). The loss of flagella during interactions with plant matrices may represent a conserved bacterial strategy to evade plant immune defenses. However, it may also reflect an energetic trade-off, as flagella are of limited functional benefit in our experimental system and energetically costly under resource-limited conditions. Previous studies have suggested that other attachment structures, such as aggregative fimbriae and fimbrial regulatory systems, may play a more relevant role in the attachment of *Salmonella* to plants (11, 12). However, competition experiments using *flhDC* and *flhA* deletion mutants revealed a reduced ability of these mutants to attach to sprouts, contrary to their TraDIS-Xpress results (11).

As with any transposon insertion sequencing study performed in a complex food matrix, several experimental trade-offs should be considered when interpreting the results. Recovery of the mutant library required removal of the food matrix and a subsequent outgrowth step to generate sufficient biomass for sequencing. Although this procedure may have influenced the relative abundance of some mutants, it was necessary to minimize matrix-derived effects during recovery and was applied identically across all experimental conditions, thereby preserving the validity of relative comparisons between sprout and no-matrix samples. Importantly, the fitness effects of selected candidates were independently confirmed through competition assays. Likewise, the use of commercially produced sprouts introduces a degree of biological variability that reflects real-world production systems. Our objective was therefore to identify robust fitness determinants whose contribution remains detectable despite such variation.

Finally, while the library did not achieve complete genome saturation, it provided broad genomic coverage and enabled the identification of key genetic elements. Overall, our study identifies key genetic and functional traits that contribute to *S. enterica* fitness on alfalfa sprouts during abusive cold storage, thereby extending insights beyond the traditionally studied serovars and focusing on a strain capable of internalization in plants (15). Although this serovar is typically host-adapted to pigs, its demonstrated ability to colonize plant tissues highlights the ecological flexibility of host-adapted serovars and underscores their potential relevance to food safety within production environments. While many identified pathways overlap with those previously reported in other *Salmonella* serovars on plant-associated matrices, this consistency underscores the presence of conserved functions essential for persistence across diverse serovars, including oxidative stress mitigation, cell envelope maintenance, and motility-associated traits. From a food safety perspective, these findings identify candidate targets for precision food safety interventions designed to limit *Salmonella* persistence during refrigerated storage under retail conditions while addressing the serovar diversity associated with fresh produce, including those less frequently detected but clinically relevant.

## Supplementary Material

Figure S1

Table S1

Table S2

**Fig. S1 (AEM00604-26-S0001.tif).** Growth comparison of the population dynamics of the *S.* Choleraesuis-11-Ga-2015 library and the wild-type strain.

**Table S1 (AEM00604-26-S0002.docx).** Primers used in the study.

**Table S2 (AEM00604-26-S0003.xlsx).** DESeq2 statistical analysis for the transposon insertion sequencing experiment of the bar-coded *S.* Choleraesuis library on alfalfa sprouts.

## Figures and Tables

**TABLE 1:** Genes with insertions resulting in a significant fitness effect in *S.* Choleraesuis on alfalfa sprouts in at least one comparison between time points^a^

SCH gene no.	Gene	Time points	Encoded protein^b^ and function
SCHChr_01977	*eda*	I vs d_3_ I vs d_5_	**Keto-hydroxyglutarate-aldolase/keto-deoxy-phosphogluconate aldolase** Carbohydrate metabolism -Pentose phosphate pathway -Glyoxylate and dicarboxylate metabolism
SCHChr_01464	*lpp1_2*	I vs d_5_	**Major outer membrane lipoprotein Lpp 1** Peptidoglycan biosynthesis and degradation, structural protein
SCHChr_01218	*fabF*	I vs d_1_ I vs d_3_ I vs d_5_	**3-Oxoacyl-[acyl-carrier-protein] synthase II** Lipid metabolism -Fatty acid biosynthesis -Biotin metabolism
SCHChr_03337	*pnp*	I vs d_1_ I vs d_3_ I vs d_5_	**Polyribonucleotide nucleotidyltransferase** RNA degradation, RNA degradosome component
SCHChr_02839	*stpA*		**DNA-binding protein StpA** HNS (histone-like nucleoid structuring)-like protein Central regulatory role in gene silencing
SCHChr_03621	*SCHChr_03621*		**Hypothetical protein** Predicted type II toxin–antitoxin system TacA family antitoxin

a Time points: I, inoculum; d_1_, 1 h post-inoculation; d_3_, 48 h post-inoculation; d_5_, 96 h post-inoculation.

b Boldface denotes the encoded protein.

**TABLE 2:** Significantly enriched KEGG pathway and associated GO terms for the interaction of *S.* Choleraesuis on alfalfa sprouts at 8°C^a^

KEGG pathway	*q* value	Selected genes	GO IDs	GO term	Classic Fisher
Cellular processes	4.72 E-04	*flgAE*	GO:0051674	Localization of the cell	8.6 E-04
		*flhC*	GO:0071973	Bacterial-type flagellum-dependent cell motility	1.0 E-03
Cell motility		*fliGK, fljB*			
Flagellar assembly			GO:0040011	Locomotion	8.6 E-03

a Listed genes fulfilled *P*_adj_ < 0.05 in at least one alfalfa sprouts/negative control and inoculum/time point comparison.

**TABLE 3:** Fitness effect of selected *S.* Choleraesuis 11-Ga-2015 individual insertion mutants in competition with the wild-type strain on alfalfa sprouts^a,d^

Condition	SCH locus tag	Gene	Gene function	Transposon assay^b^	Competition assays														
					Alfalfa sprouts					LB					PBS				
					CI sense	Stdev sense	CI antisense	stdev antisense	Outcome^c^	CI sense	Stdev sense	CI antisense	Stdev antisense	Outcome^c^	CI sense	Stdev sense	CI antisense	Stdev antisense	Outcome^c^
8°C, 48 h	SCHChr_01977	***eda***	Keto-hydroxyglutarate-aldolase/keto-deoxy-phosphogluconate aldolase	↓			**0.39**	**0.36**	↓			1.54	1.27	○			1.10	1.42	○
8°C, 48 h	SCHChr_01218	***fabF***	3-Oxoacyl-[acyl-carrier-protein] synthase II	↓	**0.45**	**0.32**			↓	**3.51**	**1.88**			↑	**3.90**	**2.81**			↑
8°C, 96 h	SCHChr_01464	lpp1_2	Major outer membrane lipoprotein Lpp 1	↓			1.51	1.52	○			2.00	1.30	○			1.51	1.22	○
8°C, 48 h	SCHChr_03926	***mnmG***	tRNA uridine 5-carboxymethy-laminomethyl modification enzyme MnmG	(↓)	**0.09**	**0.07**	**0.04**	**0.03**	↓	0.80	0.41	0.84	0.19	○	0.83	0.58	1.17	0.45	○
8°C, 48 h	SCHChr_02839	stpA	DNA-binding protein StpA	↓	2.01	1.63	**0.45**	**0.05**	↙	**7.87**	**5.94**	**2.52**	**1.41**	↑	**2.73**	**0.64**	1.10	0.43	↗

a From three to four separate experiments. Genes in bold are important for the fitness of *Salmonella* Choleraesuis on alfalfa sprouts but not in LB or PBS. $\text{CI (competition index)}=\frac{{\left(\frac{\text{mutant}}{\text{wildtype}}\right)}_{\text{timepoint}}}{{\left(\frac{\text{mutant}}{\text{wildtype}}\right)}_{\text{inoculum}}}$.

b Transposon assay data: ↓, statistically significant negative fitness effect; (), mutants with a twofold fitness effect at one OR the other criterion of the dual requirement, at *P*_adj_ ≤ 0.1. Sense: individual insertion mutant with kanamycin resistance cassette in the sense orientation of the gene; antisense: individual insertion mutant with kanamycin resistance cassette in the antisense orientation of the gene.

c Fitness effects: ↓, single-gene deletion mutants with a significant fitness disadvantage in competition assays compared to wild type (CI + stdev < 1); ↑, advantage in competition assays compared to wild type (CI − stdev > 1); ○, no effect; ↙, negative orientation-dependent effect in the antisense insertion mutant; ↖, positive orientation-dependent effect in the antisense insertion mutant; ↗, positive orientation-dependent effect in the sense insertion mutant; ↘, negative orientation-dependent effect in the sense insertion mutant.

d The bolded values indicate the classification of advantage or disadvantage compared with no observed effect, based on the calculation described in the table legend.

## Data Availability

The raw Illumina sequencing reads generated in this study are publicly available in the Open Data LMU repository under https://doi.org/10.5282/ubm/data.824. The genome sequence of *S. enterica* serovar Choleraesuis strain 11-Ga-2015 (SCH) is available in GenBank under accession no. JADWDF000000000. All remaining data are included in this article and its supplemental material.

## IMPORTANCE

Food safety strategies are frequently based on knowledge derived from well-studied, epidemiologically relevant *Salmonella* serovars, yet many less frequent types still pose a risk to consumers and may contaminate fresh produce. Different *Salmonella* serovars may vary in the relative contribution of persistence mechanisms. Recognizing these differences is essential for improving precision food safety efforts, particularly for foods such as sprouts that are repeatedly linked to outbreaks. This study highlights that less-studied serovars can rely on both shared survival strategies and unique traits that might otherwise not be captured by current control approaches. By demonstrating that strain diversity influences persistence on fresh produce, this work supports the development of precision food safety strategies that address a broader spectrum of *Salmonella*, thereby improving risk assessment and helping to better protect public health.

## References

[1] Official Journal of the European Union. 2017. ESSA hygiene guideline for the production of sprouts and seeds for sprouting (2017/C 220/03)

[2] Benincasa P, Falcinelli B, Lutts S, Stagnari F, Galieni A. 2019. Sprouted grains: a comprehensive review. Nutrients 11:421. 10.3390/nu11020421 PMC6413227 30781547

[3] Miyahira RF, Antunes AEC. 2021. Bacteriological safety of sprouts: a brief review. Int J Food Microbiol 352:109266. 10.1016/j.ijfoodmicro.2021.109266 34111728

[4] Young Kim S, Ban G-H, Won Hong Y, Ji Jang M, Ae Kim S. 2022. Microbiome shifts in sprouts (alfalfa, radish, and rapeseed) during production from seed to sprout using 16S rRNA microbiome sequencing. Food Res Int 152:110896. 10.1016/j.foodres.2021.110896 35181074

[5] Rahman M, Alam M-U, Luies SK, Kamal A, Ferdous S, Lin A, Sharior F, Khan R, Rahman Z, Parvez SM, Amin N, Hasan R, Tadesse BT, Taneja N, Islam MA, Ercumen A. 2022. Contamination of fresh produce with antibiotic-resistant bacteria and associated risks to human health: a scoping review. IJERPH 19:360. 10.3390/ijerph19010360 PMC8744955 35010620

[6] Frank C, Werber D, Cramer JP, Askar M, Faber M, an der Heiden M, Bernard H, Fruth A, Prager R, Spode A, Wadl M, Zoufaly A, Jordan S, Kemper MJ, Follin P, Müller L, King LA, Rosner B, Buchholz U, Stark K, Krause G. 2011. Epidemic profile of shiga-toxin-producing *Escherichia coli* O104:H4 outbreak in Germany. N Engl J Med 365:1771–1780. 10.1056/NEJMoa1106483 21696328

[7] Ma J, Dai J, Cao C, Su L, Cao M, He Y, Li M, Zhang Z, Chen J, Cui S, Yang B. 2024. Prevalence, serotype, antimicrobial susceptibility, contamination factors, and control methods of *Salmonella* spp. in retail fresh fruits and vegetables: a systematic review and meta-analysis. Compr Rev Food Sci Food Saf 23:e13407. 10.1111/1541-4337.13407 39030802

[8] EFSA, ECDC. 2025. The European Union One Health 2024 Zoonoses Report. EFS2 23. 10.2903/j.efsa.2025.9759 PMC12686834 41377167

[9] Caulcrick-Grimes M, Loeck BKD, Homola P, Fu T-J, Burgos J, Schoelen D, Literman R, Ladines E, Buchholz A, Tung G, 2026. An investigation of an outbreak of *Salmonella* Typhimurium infections linked to alfalfa sprouts – United States, 2022. Food Control 181:111654. 10.1016/j.foodcont.2025.111654

[10] European Food Safety Authority. 2025. Prolonged cross - border multi - serovar *Salmonella* outbreak linked to consumption of sprouted seeds. EFS3 22. 10.2903/sp.efsa.2025.EN-9315

[11] Holden ER, Abi Assaf J, Al-Khanaq H, Vimont N, Webber MA, Trampari E. 2024. Identification of pathways required for *Salmonella* to colonize alfalfa using TraDIS-Xpress Appl Environ Microbiol 90:e0013924. 10.1128/aem.00139-24 PMC11267905 38904400

[12] Barak JD, Gorski L, Naraghi-Arani P, Charkowski AO. 2005. *Salmonella enterica* virulence genes are required for bacterial attachment to plant tissue. Appl Environ Microbiol 71:5685–5691. 10.1128/AEM.71.10.5685-5691.2005 PMC1265987 16204476

[13] Jajere SM. 2019. A review of *Salmonella enterica* with particular focus on the pathogenicity and virulence factors, host specificity and antimicrobial resistance including multidrug resistance. Vet World 12:504–521. 10.14202/vetworld.2019.504-521 PMC6515828 31190705

[14] Evans EW, Redmond EC. 2015. Analysis of older adults’ domestic kitchen storage practices in the United Kingdom: identification of risk factors associated with listeriosis. J Food Prot 78:738–745. 10.4315/0362-028X.JFP-14-527 25836399

[15] Esteban-Cuesta I, Drees N, Ulrich S, Stauch P, Sperner B, Schwaiger K, Gareis M, Gottschalk C. 2018. Endogenous microbial contamination of melons (*Cucumis melo*) from international trade: an underestimated risk for the consumer? J Sci Food Agric 98:5074–5081. 10.1002/jsfa.9045 29604072

[16] Jones TF, Ingram LA, Cieslak PR, Vugia DJ, Tobin-D’Angelo M, Hurd S, Medus C, Cronquist A, Angulo FJ. 2008. Salmonellosis outcomes differ substantially by serotype. J Infect Dis 198:109–114. 10.1086/588823 18462137

[17] Esteban-Cuesta I, Fischer J, Guldimann C. 2021. Draft genome sequence of a *Salmonella enterica* subsp. enterica serotype Choleraesuis strain isolated from the pulp of muskmelons. Microbiol Resour Announc 10:e00009–21. 10.1128/MRA.00009-21 PMC7953279 33707316

[18] de Moraes MH, Desai P, Porwollik S, Canals R, Perez DR, Chu W, McClelland M, Teplitski M. 2017. Salmonella persistence in tomatoes requires a distinct set of metabolic functions identified by transposon insertion sequencing. Appl Environ Microbiol 83:e03028–16. 10.1128/AEM.03028-16 PMC5311394 28039131

[19] de Moraes MH, Soto EB, Salas González I, Desai P, Chu W, Porwollik S, McClelland M, Teplitski M. 2018. Genome-wide comparative functional analyses reveal adaptations of *Salmonella* sv. newport to a plant colonization lifestyle. Front Microbiol 9:877. 10.3389/fmicb.2018.00877 PMC5968271 29867794

[20] Irene Esteban-Cuesta, Führer L, Porwollik S, Chu W, Fiddaman SR, Sah I, McClelland M, Guldimann C. 2026. Genomic factors contributing to the resilience of *Salmonella enterica* on ready-to-eat muskmelon. Food Microbiol 134:104947. 10.1016/j.fm.2025.104947 PMC12767474 41136164

[21] Führer L, Porwollik S, Chu W, Hohenester V, Sah I, McClelland M, Guldimann C, Esteban-Cuesta I. 2026. A screen of *Salmonella enterica* mutants interacting with fresh onions and alfalfa sprouts. Int J Food Microbiol 453:111696. 10.1016/j.ijfoodmicro.2026.111696 PMC13152249 41831350

[22] Love MI, Huber W, Anders S. 2014. Moderated estimation of fold change and dispersion for RNA-seq data with DESeq2. Genome Biol 15:550. 10.1186/s13059-014-0550-8 PMC4302049 25516281

[23] Kanehisa M, Furumichi M, Sato Y, Kawashima M, Ishiguro-Watanabe M. 2023. KEGG for taxonomy-based analysis of pathways and genomes. Nucleic Acids Res 51:D587–D592. 10.1093/nar/gkac963 PMC9825424 36300620

[24] Aleksander SA, Balhoff J, Carbon S, Cherry JM, Drabkin HJ, Ebert D, Feuermann M, Gaudet P, Harris NL, Hill DP, 2023. The gene ontology consortium. the gene ontology knowledgebase in 2023. Genetics 224. 10.1093/genetics/iyad031 PMC10158837 36866529

[25] Ashburner M, Ball CA, Blake JA, Botstein D, Butler H, Cherry JM, Davis AP, Dolinski K, Dwight SS, Eppig JT, Harris MA, Hill DP, Issel-Tarver L, Kasarskis A, Lewis S, Matese JC, Richardson JE, Ringwald M, Rubin GM, Sherlock G. 2000. Gene ontology: tool for the unification of biology. Nat Genet 25:25–29. 10.1038/75556 PMC3037419 10802651

[26] Thomas PD, Ebert D, Muruganujan A, Mushayahama T, Albou L-P, Mi H. 2022. PANTHER: making genome-scale phylogenetics accessible to all. Protein Sci 31:8–22. 10.1002/pro.4218 PMC8740835 34717010

[27] Alexa A, Rahnenfuhrer J. 2024. topGO: enrichment analysis for gene ontology. https://bioconductor.org/packages/topGO.

[28] Wu T, Hu E, Xu S, Chen M, Guo P, Dai Z, Feng T, Zhou L, Tang W, Zhan L, Fu X, Liu S, Bo X, Yu G. 2021. clusterProfiler 4.0: a universal enrichment tool for interpreting omics data. Innovation (Camb) 2:100141. 10.1016/j.xinn.2021.100141 PMC8454663 34557778

[29] Monge Brenes AL, Brown W, Steinmaus S, Brecht JK, Xie Y, Bornhorst ER, Luo Y, Zhou B, Shaw A, Vorst K. 2020. Temperature profiling of open- and closed-doored produce cases in retail grocery stores. Food Control 113:107158. 10.1016/j.foodcont.2020.107158

[30] Jang MJ, Kim SY, Ricke SC, Rhee MS, Kim SA. 2021. Microbial ecology of alfalfa, radish, and rapeseed sprouts based on culture methods and 16S rRNA microbiome sequencing. Food Res Int 144:110316. 10.1016/j.foodres.2021.110316 34053521

[31] Law RC, Nurwono G, Park JO. 2024. A parallel glycolysis provides a selective advantage through rapid growth acceleration. Nat Chem Biol 20:314–322. 10.1038/s41589-023-01395-2 PMC10987256 37537378

[32] Mitosch K, Beyß M, Phapale P, Drotleff B, Nöh K, Alexandrov T, Patil KR, Typas A. 2023. A pathogen-specific isotope tracing approach reveals metabolic activities and fluxes of intracellular *Salmonella*. PLoS Biol 21:e3002198. 10.1371/journal.pbio.3002198 PMC10468081 37594988

[33] Eriksson S, Lucchini S, Thompson A, Rhen M, Hinton JCD. 2003. Unravelling the biology of macrophage infection by gene expression profiling of intracellular *Salmonella enterica*. Mol Microbiol 47:103–118. 10.1046/j.1365-2958.2003.03313.x 12492857

[34] Clements MO, Eriksson S, Thompson A, Lucchini S, Hinton JCD, Normark S, Rhen M. 2002. Polynucleotide phosphorylase is a global regulator of virulence and persistency in *Salmonella enterica*. Proc Natl Acad Sci USA 99:8784–8789. 10.1073/pnas.132047099 PMC124376 12072563

[35] Li Y, Salazar JK, He Y, Desai P, Porwollik S, Chu W, Paola P-SS, Tortorello ML, Juarez O, Feng H, McClelland M, Zhang W. 2020. Mechanisms of *Salmonella* attachment and survival on in-shell black peppercorns, almonds, and hazelnuts. Front Microbiol 11. 10.3389/fmicb.2020.582202 PMC7644838 33193218

[36] Guest RL, Rutherford ST, Silhavy TJ. 2021. Border control: regulating LPS biogenesis. Trends Microbiol 29:334–345. 10.1016/j.tim.2020.09.008 PMC7969359 33036869

[37] Ricke SC, Dawoud TM, Kim SA, Park SH, Kwon YM. 2018. *Salmonella* cold stress response: mechanisms and occurrence in foods. Adv Appl Microbiol 104:1–38. 10.1016/bs.aambs.2018.03.001 30143250

[38] Garwin JL, Klages AL, Cronan JE Jr. 1980. Beta-ketoacyl-acyl carrier protein synthase II of *Escherichia coli*. Evidence for function in the thermal regulation of fatty acid synthesis. J Biol Chem 255:3263–3265. 10.1016/S0021-9258(19)85692-2 6988423

[39] Zhu L, Cheng J, Luo B, Feng S, Lin J, Wang S, Cronan JE, Wang H. 2009. Functions of the *Clostridium acetobutylicium* FabF and FabZ proteins in unsaturated fatty acid biosynthesis. BMC Microbiol 9:119. 10.1186/1471-2180-9-119 PMC2700279 19493359

[40] Allen EE, Bartlett DH. 2000. FabF is required for piezoregulation of cis-vaccenic acid levels and piezophilic growth of the deep-sea bacterium *Photobacterium profundum* strain SS9. J Bacteriol 182:1264–1271. 10.1128/JB.182.5.1264-1271.2000 PMC94411 10671446

[41] Jayeola V, McClelland M, Porwollik S, Chu W, Farber J, Kathariou S. 2020. Identification of novel genes mediating survival of salmonella on low-moisture foods via transposon sequencing analysis. Front Microbiol 11:726. 10.3389/fmicb.2020.00726 PMC7242855 32499760

[42] Scherber CM, Schottel JL, Aksan A. 2009. Membrane phase behavior of *Escherichia coli* during desiccation, rehydration, and growth recovery. Biochim Biophys Acta 1788:2427–2435. 10.1016/j.bbamem.2009.08.011 19716799

[43] Sha J, Fadl AA, Klimpel GR, Niesel DW, Popov VL, Chopra AK. 2004. The two murein lipoproteins of *Salmonella enterica* serovar Typhimurium contribute to the virulence of the organism. Infect Immun 72:3987–4003. 10.1128/IAI.72.7.3987-4003.2004 PMC427434 15213144

[44] Zhou D, Wei X, Jin Y, Cheng Z, Jin S, Ha U-H, Pan X, Wu W. 2025. The fatty acid synthesis gene. Microbiol Spectr 13:e01339–25. 10.1128/spectrum.01339-25 PMC12323585 40662729

[45] Szczepaniak J, Webby MN. 2024. The tol pal system integrates maintenance of the three layered cell envelope. NPJ Antimicrob Resist 2:46. 10.1038/s44259-024-00065-0 PMC11721397 39843782

[46] Shippy DC, Fadl AA. 2014. tRNA modification enzymes GidA and MnmE: potential role in virulence of bacterial pathogens. Int J Mol Sci 15:18267–18280. 10.3390/ijms151018267 PMC4227215 25310651

[47] Shippy DC, Heintz JA, Albrecht RM, Eakley NM, Chopra AK, Fadl AA. 2012. Deletion of glucose-inhibited division (gidA) gene alters the morphological and replication characteristics of *Salmonella enterica Serovar typhimurium*. Arch Microbiol 194:405–412. 10.1007/s00203-011-0769-7 22109813

[48] Ahmad S, Jose da Costa Gonzales L, Bowler-Barnett EH, Rice DL, Kim M, Wijerathne S, Luciani A, Kandasaamy S, Luo J, Watkins X, Turner E, Martin MJ, Consortium tU. 2025. The UniProt website API: facilitating programmatic access to protein knowledge. Nucleic Acids Res 53:W547–W553. 10.1093/nar/gkaf394 PMC12230682 40331428

[49] Camacho C, Coulouris G, Avagyan V, Ma N, Papadopoulos J, Bealer K, Madden TL. 2009. BLAST+: architecture and applications. BMC Bioinformatics 10:421. 10.1186/1471-2105-10-421 PMC2803857 20003500

[50] Maisonneuve E, Gerdes K. 2014. Molecular mechanisms underlying bacterial persisters. Cell 157:539–548. 10.1016/j.cell.2014.02.050 24766804

[51] Helaine S, Kugelberg E. 2014. Bacterial persisters: formation, eradication, and experimental systems. Trends Microbiol 22:417–424. 10.1016/j.tim.2014.03.008 24768561

[52] Cheverton Angela M, Gollan B, Przydacz M, Wong Chi T, Mylona A, Hare Stephen A, Helaine S. 2016. A salmonella toxin promotes persister formation through acetylation of tRNA. Mol Cell 63:86–96. 10.1016/j.molcel.2016.05.002 PMC4942678 27264868

[53] Li Y, Salazar JK, He Y, Desai P, Porwollik S, Chu W, Paola P-S, Tortorello ML, Juarez O, Feng H, McClelland M, Zhang W. 2020. Mechanisms of *Salmonella* attachment and survival on in-shell black peppercorns, almonds, and hazelnuts. Front Microbiol 11:582202. 10.3389/fmicb.2020.582202 PMC7644838 33193218

[54] Gorski L, Duhé JM, Flaherty D. 2009. The use of flagella and motility for plant colonization and fitness by different strains of the foodborne pathogen *Listeria monocytogenes*. PLoS One 4:e5142. 10.1371/journal.pone.0005142 PMC2664462 19357783

