# Supplementary material for: Identification of genes promoting fitness of a plant-associated Salmonella Choleraesuis strain on alfalfa sprouts during cold storage
Source: Appl Environ Microbiol. Author manuscript; Available in PMC 2026 Sep 25. (PMC13599476; doi:10.1128/aem.00604-26)
Supplement: Supplementary file 1 [file NIHMS2209049-supplement-Table_S1.docx]

**Supplementary Table 1.** Primers used in the study.

| **Primer Name** | **Primer Sequence** |
| --- | --- |
| Tn*5*PCR1L | CTGTCTCTTATACACATCTCAACCATCATC |
| Tn5PCR1R | CTGTCTCTTATACACATCTCAACCCTG |
| 5P-Tn5PCR2L | P-CTGTCTCTTATACACATCTCAACCATCA |
| 5P-Tn5PCR2R | CTGTCTCTTATACACATCTCAACCCTGCACTCTTTCCCTACACGACGC  TCTTCCGATCT-N21-AGATCGGAAGAGCACACGTCTGAACTCCAGTA  GGCCGGTCTCCCTATAGTGAGTCGTATTAATTTCGATAAG |
| KlenowN_10_ | CACCCACAAACCAACACCCCACAAC-N10 |
| Klenow_PCR1SCHL | CACCCACAAACCAACACCCCACAAC |
| Klenow_PCR1SCHR | GTGACTGGAGTTCAGACGTGTGCTCTTCCGA |
| Klenow_PCR2SCHL | AATGATACGGCGACCACCGAGATCTACACCCACAAACCAACACCCC  ACAAC |
| PCRSCHoTnL | AATGATACGGCGACCACCGAGATCTACAC-N8-ACACTCTTTCCCTAC  ACGACGCTCTTCCGATCT |
| PCRSCHoTnR | CAAGCAGAAGACGGCATACGAGAT-N8-GTGACTGGAGTTCAGACGT  GTGCTCTTCCGATCT |
| N701_pCLF_right_read1_TCGCCTTA | AATGATACGGCGACCACCGAGATCTACACTCGCCTTAACACTCTTTC  CCTACACGACGCTCTTCCGATCT |
| N702_pCLF_right_read1_CTAGTACG | AATGATACGGCGACCACCGAGATCTACACCTAGTACGACACTCTTTC  CCTACACGACGCTCTTCCGATCT |
| N703_pCLF_right_read1_TTCTGCCT | AATGATACGGCGACCACCGAGATCTACACTTCTGCCTACACTCTTTC  CCTACACGACGCTCTTCCGATCT |
| N704_pCLF_right_read1_GCTCAGGA | AATGATACGGCGACCACCGAGATCTACACGCTCAGGAACACTCTTTC  CCTACACGACGCTCTTCCGATCT |
| N705_pCLF_right_read1_AGGAGTCC | AATGATACGGCGACCACCGAGATCTACACAGGAGTCCACACTCTTTC  CCTACACGACGCTCTTCCGATCT |
| N706_pCLF_right_read1_CATGCCTA | AATGATACGGCGACCACCGAGATCTACACCATGCCTAACACTCTTTC  CCTACACGACGCTCTTCCGATCT |
| N707_pCLF_right_read1_GTAGAGAG | AATGATACGGCGACCACCGAGATCTACACGTAGAGAGACACTCTTTC  CCTACACGACGCTCTTCCGATCT |
| N708_pCLF_right_read1_CCTCTCTG | AATGATACGGCGACCACCGAGATCTACACCCTCTCTGACACTCTTTC  CCTACACGACGCTCTTCCGATCT |
| N709_pCLF_right_read1_AGCGTAGC | AATGATACGGCGACCACCGAGATCTACACAGCGTAGCACACTCTTTC  CCTACACGACGCTCTTCCGATCT |
| N710_pCLF_right_read1_CAGCCTCG | AATGATACGGCGACCACCGAGATCTACACCAGCCTCGACACTCTTTC  CCTACACGACGCTCTTCCGATCT |
| N711_pCLF_right_read1_TGCCTCTT | AATGATACGGCGACCACCGAGATCTACACTGCCTCTTACACTCTTTC  CCTACACGACGCTCTTCCGATCT |
| N712_pCLF_right_read1_TCCTCTAC | AATGATACGGCGACCACCGAGATCTACACTCCTCTACACACTCTTTC  CCTACACGACGCTCTTCCGATCT |
| S503_pCLF_right_read2_TATCCTCT | CAAGCAGAAGACGGCATACGAGATTATCCTCTGTGACTGGAGTTC  AGACGTGTGCTCTTCCGATCT |
| S505_pCLF_right_read2_GTAAGGAG | CAAGCAGAAGACGGCATACGAGATGTAAGGAGGTGACTGGAGTTC  AGACGTGTGCTCTTCCGATCT |
| S506_pCLF_right_read2_ACTGCATA | CAAGCAGAAGACGGCATACGAGATACTGCATAGTGACTGGAGTTC  AGACGTGTGCTCTTCCGATCT |
| S507_pCLF_right_read2_AAGGAGTA | CAAGCAGAAGACGGCATACGAGATAAGGAGTAGTGACTGGAGTTC  AGACGTGTGCTCTTCCGATCT |
| S508_pCLF_right_read2_CTAAGCCT | CAAGCAGAAGACGGCATACGAGATCTAAGCCTGTGACTGGAGTTC  AGACGTGTGCTCTTCCGATCT |
| S510_pCLF_right_read2_CGTCTAAT | CAAGCAGAAGACGGCATACGAGATCGTCTAATGTGACTGGAGTTC  AGACGTGTGCTCTTCCGATCT |
| S511_pCLF_right_read2_TCTCTCCG | CAAGCAGAAGACGGCATACGAGATTCTCTCCGGTGACTGGAGTTC  AGACGTGTGCTCTTCCGATCT |
| S513_pCLF_right_read2_TCGACTAG | CAAGCAGAAGACGGCATACGAGATTCGACTAGGTGACTGGAGTTC  AGACGTGTGCTCTTCCGATCT |
| S515_pCLF_right_read2_TTCTAGCT | CAAGCAGAAGACGGCATACGAGATTTCTAGCTGTGACTGGAGTTC  AGACGTGTGCTCTTCCGATCT |
| S516_pCLF_right_read2_CCTAGAGT | CAAGCAGAAGACGGCATACGAGATCCTAGAGTGTGACTGGAGTTC  AGACGTGTGCTCTTCCGATCT |
| S517_pCLF_right_read2_GCGTAAGA | CAAGCAGAAGACGGCATACGAGATGCGTAAGAGTGACTGGAGTTC  AGACGTGTGCTCTTCCGATCT |
