# Supplementary material for: Identification of genes promoting fitness of a plant-associated Salmonella Choleraesuis strain on alfalfa sprouts during cold storage
Source: Appl Environ Microbiol. 2026 Aug 10;92(9):e00604-26. doi: 10.1128/aem.00604-26 (PMC13599476; doi:10.1128/aem.00604-26)
Supplement: Supplementary file 1 [file aem.00604-26-s0002.docx]

**Supplementary Table 1.** Primers used in the study.

| **Primer Name** | **Primer Sequence** |
| --- | --- |
| Tn*5*PCR1L | CTGTCTCTTATACACATCTCAACCATCATC |
| Tn5PCR1R | CTGTCTCTTATACACATCTCAACCCTG |
| 5P-Tn5PCR2L | P-CTGTCTCTTATACACATCTCAACCATCA |
| 5P-Tn5PCR2R | CTGTCTCTTATACACATCTCAACCCTGCACTCTTTCCCTACACGACGC TCTTCCGATCT-N21-AGATCGGAAGAGCACACGTCTGAACTCCAGTA GGCCGGTCTCCCTATAGTGAGTCGTATTAATTTCGATAAG |
| KlenowN_10_ | CACCCACAAACCAACACCCCACAAC-N10 |
| Klenow_PCR1SCHL | CACCCACAAACCAACACCCCACAAC |
| Klenow_PCR1SCHR | GTGACTGGAGTTCAGACGTGTGCTCTTCCGA |
| Klenow_PCR2SCHL | AATGATACGGCGACCACCGAGATCTACACCCACAAACCAACACCCC ACAAC |
| PCRSCHoTnL | AATGATACGGCGACCACCGAGATCTACAC-N8-ACACTCTTTCCCTAC ACGACGCTCTTCCGATCT |
| PCRSCHoTnR | CAAGCAGAAGACGGCATACGAGAT-N8-GTGACTGGAGTTCAGACGT GTGCTCTTCCGATCT |
| N701_pCLF_right_read1_TCGCCTTA | AATGATACGGCGACCACCGAGATCTACACTCGCCTTAACACTCTTTC CCTACACGACGCTCTTCCGATCT |
| N702_pCLF_right_read1_CTAGTACG | AATGATACGGCGACCACCGAGATCTACACCTAGTACGACACTCTTTC CCTACACGACGCTCTTCCGATCT |
| N703_pCLF_right_read1_TTCTGCCT | AATGATACGGCGACCACCGAGATCTACACTTCTGCCTACACTCTTTC CCTACACGACGCTCTTCCGATCT |
| N704_pCLF_right_read1_GCTCAGGA | AATGATACGGCGACCACCGAGATCTACACGCTCAGGAACACTCTTTC CCTACACGACGCTCTTCCGATCT |
| N705_pCLF_right_read1_AGGAGTCC | AATGATACGGCGACCACCGAGATCTACACAGGAGTCCACACTCTTTC CCTACACGACGCTCTTCCGATCT |
| N706_pCLF_right_read1_CATGCCTA | AATGATACGGCGACCACCGAGATCTACACCATGCCTAACACTCTTTC CCTACACGACGCTCTTCCGATCT |
| N707_pCLF_right_read1_GTAGAGAG | AATGATACGGCGACCACCGAGATCTACACGTAGAGAGACACTCTTTC CCTACACGACGCTCTTCCGATCT |
| N708_pCLF_right_read1_CCTCTCTG | AATGATACGGCGACCACCGAGATCTACACCCTCTCTGACACTCTTTC CCTACACGACGCTCTTCCGATCT |
| N709_pCLF_right_read1_AGCGTAGC | AATGATACGGCGACCACCGAGATCTACACAGCGTAGCACACTCTTTC CCTACACGACGCTCTTCCGATCT |
| N710_pCLF_right_read1_CAGCCTCG | AATGATACGGCGACCACCGAGATCTACACCAGCCTCGACACTCTTTC CCTACACGACGCTCTTCCGATCT |
| N711_pCLF_right_read1_TGCCTCTT | AATGATACGGCGACCACCGAGATCTACACTGCCTCTTACACTCTTTC CCTACACGACGCTCTTCCGATCT |
| N712_pCLF_right_read1_TCCTCTAC | AATGATACGGCGACCACCGAGATCTACACTCCTCTACACACTCTTTC CCTACACGACGCTCTTCCGATCT |
| S503_pCLF_right_read2_TATCCTCT | CAAGCAGAAGACGGCATACGAGATTATCCTCTGTGACTGGAGTTC AGACGTGTGCTCTTCCGATCT |
| S505_pCLF_right_read2_GTAAGGAG | CAAGCAGAAGACGGCATACGAGATGTAAGGAGGTGACTGGAGTTC AGACGTGTGCTCTTCCGATCT |
| S506_pCLF_right_read2_ACTGCATA | CAAGCAGAAGACGGCATACGAGATACTGCATAGTGACTGGAGTTC AGACGTGTGCTCTTCCGATCT |
| S507_pCLF_right_read2_AAGGAGTA | CAAGCAGAAGACGGCATACGAGATAAGGAGTAGTGACTGGAGTTC AGACGTGTGCTCTTCCGATCT |
| S508_pCLF_right_read2_CTAAGCCT | CAAGCAGAAGACGGCATACGAGATCTAAGCCTGTGACTGGAGTTC AGACGTGTGCTCTTCCGATCT |
| S510_pCLF_right_read2_CGTCTAAT | CAAGCAGAAGACGGCATACGAGATCGTCTAATGTGACTGGAGTTC AGACGTGTGCTCTTCCGATCT |
| S511_pCLF_right_read2_TCTCTCCG | CAAGCAGAAGACGGCATACGAGATTCTCTCCGGTGACTGGAGTTC AGACGTGTGCTCTTCCGATCT |
| S513_pCLF_right_read2_TCGACTAG | CAAGCAGAAGACGGCATACGAGATTCGACTAGGTGACTGGAGTTC AGACGTGTGCTCTTCCGATCT |
| S515_pCLF_right_read2_TTCTAGCT | CAAGCAGAAGACGGCATACGAGATTTCTAGCTGTGACTGGAGTTC AGACGTGTGCTCTTCCGATCT |
| S516_pCLF_right_read2_CCTAGAGT | CAAGCAGAAGACGGCATACGAGATCCTAGAGTGTGACTGGAGTTC AGACGTGTGCTCTTCCGATCT |
| S517_pCLF_right_read2_GCGTAAGA | CAAGCAGAAGACGGCATACGAGATGCGTAAGAGTGACTGGAGTTC AGACGTGTGCTCTTCCGATCT |
